# Clinical Characteristics of Concomitant Systemic Lupus Erythematosus and Primary Biliary Cirrhosis: A Literature Review

**DOI:** 10.1155/2015/713728

**Published:** 2015-05-18

**Authors:** Toru Shizuma

**Affiliations:** Department of Physiology, School of Medicine, Tokai University, 143 Shimokasuya, Isehara, Kanagawa 259-1193, Japan

## Abstract

Although autoimmune diseases often coexist, concomitant cases of systemic lupus erythematosus (SLE) and primary biliary cirrhosis (PBC) are uncommon. In this review paper, 34 cases of SLE with concomitant PBC found in English and Japanese scientific literature and Japanese proceedings were reviewed and summarized, including cases with liver dysfunction complicated by SLE. Of the 34 reported concomitant cases of SLE and PBC, 97.1% (33/34) were females, and PBC was diagnosed initially in 69.0% (20/29), except for five cases in which both SLE and PBC were simultaneously diagnosed. Sjögren's syndrome was the most common autoimmune disease complicating concomitant SLE and PBC (23.5%, 8/34). Five deaths have been reported: two elderly patients died of liver failure because of the worsening of PBC, and another two patients died from pulmonary infection associated with SLE pharmacotherapy. It is uncertain whether concomitant cases occur by chance or share a common immunological or genetic basis.

## 1. Introduction

Autoimmune diseases exhibit an increased immune response to self-antigens and predominantly occur in females [1]. Autoimmune diseases share some similar pathological pathways or genetic etiologies, and it is common that more than one autoimmune condition may occur in a single patient [2].

Systemic lupus erythematosus (SLE) is a multisystem autoimmune disease that results from a combination of genetic, environmental, and hormonal factors [3–5]. It is characterized by the presence of pathogenic autoantibodies such as anti-double stranded DNA or anti-histones and immune complexes in the serum and target tissues, inducing serious inflammatory conditions by the activation of the complement system [3, 4]. The pathogenesis of SLE is mainly due to the deficiency of several immunological mechanisms, including inappropriate function of the innate immune system, altered self-tolerance mechanisms, and apoptotic cell clearance [4, 5]. Furthermore, SLE often coexists with other autoimmune diseases or collagen disorders, such as rheumatoid arthritis (RA) and Sjögren's syndrome (SjS) [6, 7].

Conversely, the liver is a target of autoimmune reactions, as observed in autoimmune diseases, such as autoimmune hepatitis (AIH), primary biliary cirrhosis (PBC), and primary sclerosing cholangitis (PSC). The liver involvement in SLE patients includes several liver diseases or SLE itself, and it is necessary to discriminate between the causes of liver dysfunction or abnormal liver enzyme values. However, it is sometimes challenging to discriminate between the causes of the liver involvement in SLE patients, and the use of immunosuppressive therapy for SLE may distort the clinical and laboratory findings of underlying autoimmune liver disease, leading to diagnostic delay or occult progression of liver cirrhosis (LC) [8].

PBC is considered an autoimmune disease of unknown etiology with organ-specific disturbance characterized by chronic progressive cholestasis with the destruction of the intrahepatic small bile ducts, particularly the interlobular bile ducts [9–13]. The causes of PBC appear to involve environmental and genetic factors [14, 15]. Moreover, it is well recognized that PBC is an overlapping condition between the autoimmune hepatology and rheumatology [16].

Reported concomitant cases of SLE and PBC are relatively rare [8, 9, 17–32]. Some patients with SLE and PBC may have a common genetic susceptibility toward developing these diseases [33]; however, it is also thought that the coincidence of SLE and PBC is purely incidental.

Currently, it is uncertain whether concomitant SLE and PBC occurs by chance or whether this has a shared common immunological and/or genetic basis.

To date, there have been few systematic literature reviews of concomitant cases of SLE and PBC, and the clinical features and pathophysiology of concomitant cases of SLE and PBC remain unclear. However, it may be necessary to discriminate between the complications of PBC in SLE patients with liver involvement because some PBC patients may progress to occult end-stage liver failure, and prolonged use of glucocorticosteroids for SLE is potentially associated with significant adverse effects, including osteoporosis, which ranges from 20% to 44% in PBC patients, increasing with the progression of PBC [34].

In this report, a literature search and a review of cases of SLE with concomitant PBC, including autoimmune liver diseases, were conducted to clarify the clinical features of concomitant SLE and PBC cases.

## 2. Methods

A literature search on scientific articles in English and Japanese and Japanese proceedings was conducted using the PubMed (http://www.ncbi.nlm.nih.gov/pubmed/) and Japana Centra Revuo Medicina (Igaku Chou Zasshi) (http://edb.kulib.kyoto-u.ac.jp/dbe/JA.html) databases, respectively, retrieving cases of concomitant SLE and PBC published to date.

Suspected cases of SLE and those of drug-induced lupus-like syndromes were excluded. Moreover, the case report by Tunccan et al. [35], which is a case of leishmaniasis with clinical and laboratory features mimicking SLE and autoimmune liver disease, was also excluded from this analysis, because autoimmune reactions induced by leishmaniasis are common, and autoimmune reactions were indeed thought to be induced by leishmaniasis in this particular case [35]. Cases of SLE diagnosed according to the American Rheumatism Association criteria were included, with patients required to have at least 4/11 criteria for receiving a diagnosis of SLE [36].

### 2.1. Liver Dysfunction and Abnormal Liver Enzyme Values in Patients with SLE

Liver dysfunction is not considered as the main organ pathology in SLE [37, 38], and liver function abnormalities are not included in the classification and diagnostic criteria of SLE [36, 38]. The frequency of liver dysfunction or abnormal liver enzyme values during the course of SLE ranged from 19% to 60% [37–49]. Moreover, the prevalence of hepatomegaly in SLE patients ranged from 12% to 55% [48, 49]. Fluctuations in alanine transaminase values corresponding to SLE activity have been reported in some patients with SLE [45, 49]; however, no correlation between SLE activity and the incidence of liver disease was identified in any of the reviewed cases [37, 47]. This discrepancy may be attributed to the different causes of liver dysfunction or abnormal liver enzyme values in SLE patients.

Several authors have suggested a role for SLE in triggering an often subclinical hepatopathy, referred to as lupus hepatitis (SLE-related hepatitis), describing this disease as hypertransaminasemia, frequently associated with exacerbation of the lupus disease, which returns to normal values after glucocorticosteroid therapy [49]. Lupus hepatitis is a distinct manifestation in 3–9% of SLE patients [45, 49, 50]. In cases of lupus hepatitis, the fluctuations in alanine transaminase values occur parallel to the activity of SLE and are generally subclinical, with few cases progressing to end-stage liver disease [49, 50]. Conversely, no obvious correlation between SLE activity and the incidence of liver disease has been identified in liver involvement except lupus hepatitis [37, 47].

Hence, the identification of the etiology of liver dysfunction in SLE (other than lupus hepatitis) is often difficult [38, 47] because of the existence of several potential causes, including AIH, PBC, hepatic steatosis, nonalcoholic fatty liver disease, viral hepatitis, and drug-induced liver diseases such as those induced by glucocorticosteroids [42, 44, 47, 51]. The prevalence of drug-induced hepatitis is relatively high in SLE patients [49], and Takahashi et al. [44, 47] suggested that drug-induced hepatitis may be a common cause (approximately 30%) of liver dysfunction in SLE patients in Japan. Statistical analyses indicate that exposure to large doses of glucocorticosteroids is a significant factor in the etiology of severe fatty liver disease [52]. The causes of drug-induced hepatitis often include therapeutic drugs for SLE, such as nonsteroidal anti-inflammatory drugs, methotrexate, azathioprine, and other nonglucocorticosteroids [49].

In addition, other reports have revealed that liver dysfunction is not a major prognostic factor of SLE [42, 45, 47, 53]. Large multicenter studies focusing on the mortality in SLE have shown that liver disease does not influence morbidity or mortality in SLE patients [53]. One of the possible explanations for this may be that end-stage liver dysfunction with concomitant SLE is generally rare [2, 38, 39, 45]. A review by Matsumoto et al. [52] revealed that LC was evident in only 1.1% of liver biopsies from 1,468 Japanese patients with SLE.

### 2.2. Characteristics of PBC

#### 2.2.1. Pathogenesis of PBC

Although the etiology and pathogenesis of PBC remain largely unknown, there is an increasing evidence of interplay of genetic associations, such as specific human leukocyte antigen alleles and environmental factors, in individual host susceptibility [54, 55]. PBC is characterized by the selective destruction of intrahepatic cholangiocytes [54], and PBC results from an immunologic response toward an immunodominant mitochondrial autoantigen, the E2 component of the pyruvate dehydrogenase complex (PDC-E2) [54]. The characteristics of the disease also include the presence of disease-specific anti-mitochondrial autoantibodies (AMAs) and autoreactive lymphocytes. Associations with the genetic background and environmental exposure and other suspected agents, such as xenobiotics and microbes, appear to influence PBC by facilitating molecular mimicry, leading to loss of tolerance to PDC-E2, development of AMA, and subsequent development of autoimmunity and subsequent biliary injury in PBC [55, 56].

#### 2.2.2. Clinical Features of PBC

PBC affects middle-aged (penta- and sexagenarians) women much more commonly than men (female : male ratio, 9-10 : 1) [11–13, 15, 34]. Its prevalence is 19–402 cases per million [13, 15, 34]. Comorbidity with other autoimmune disease, familial incidence, past or present smoking habits, a history of urinary tract infection, and repetitive use of cosmetic products such as hair dyes are significant risk factors for developing PBC [13, 14, 16].

The clinical features and natural history of PBC range significantly from asymptomatic to progressive conditions [13]. Jaundice, pruritus derived from cholestasis, and general fatigue are typical symptoms in patients with PBC. However, up to 60% of patients may have no clinical symptoms (asymptomatic PBC). PBC represents approximately 1% of all cases of LC [34], potentially reflecting the relatively high frequency of asymptomatic cases.

#### 2.2.3. Histopathological Features of PBC

With regard to the histopathological features of PBC, florid bile duct lesions, such as chronic, nonsuppurative, and destructive cholangitis, and epithelioid granuloma formation are well-known and useful histological findings in the diagnosis of PBC [13]. Other histopathological findings include portal inflammation, chronic cholestasis, hepatic changes (interface hepatitis or lobular hepatitis), and bile duct loss. The classifications by Scheuer or Ludwig are globally used for disease staging and are based on the histopathological findings of PBC.

#### 2.2.4. Diagnosis of PBC

The diagnosis of PBC is established if two of the three objective criteria are present: (1) elevated serum alkaline phosphatase; (2) presence of AMA, which is useful for the serological diagnosis of PBC (90%–95% of patients with PBC being AMA-positive [25, 57]); and (3) liver histology findings (presence of chronic, nonsuppurative, and destructive cholangitis) [13].

#### 2.2.5. Prognosis of PBC

The prognosis of PBC is often dependent on the development of portal hypertension or cirrhosis, indicating liver failure. However, disease progression may in some cases be significantly inhibited by treatment with ursodeoxycholic acid (UDCA) [13, 58]. Meanwhile, patients with end-stage liver failure require organ transplantation [34]. In such cases, prognostic models, such as Mayo risk scores and bilirubin levels, are useful to determine the appropriate timing of liver transplantation [58]. Although the incidence of hepatocellular carcinoma (HCC) with concomitant PBC is relatively low, several studies have reported incidence rates of <1.6% [9, 59, 60]. Floreani et al. [61] reported that the prevalence of HCC in patients with PBC was 3.0% (11/361) after a mean follow-up period of 8 ± 6.9 years. According to Harada and Nakanuma [10], the incidence of HCC in patients with PBC has increased over the recent decades in Japan. The development of HCC may be associated with refractory to UDCA [58].

#### 2.2.6. PBC/AIH Overlap

PBC/AIH overlap is a relatively rare condition, affecting <10% of patients with AIH or PBC [62, 63]. Recently, Efe et al. [64] reported that, among 1,065 patients diagnosed with PBC (*n* = 483) and AIH (*n* = 582), a progressive development of PBC-AIH after a mean of 6.5 years of follow-up was observed in 19 patients (1.8%). Moreover, the combination of UDCA and immunosuppression appears to be an appropriate therapy in cases of PBC-AIH overlap [64].

#### 2.2.7. Extrahepatic Autoimmune Disease Complicated by PBC

SjS appears to be the most common autoimmune disorder concomitantly presenting with PBC [18, 22, 24, 65]. Similarly, RA, systemic sclerosis (SS), Raynaud's syndrome, and chronic thyroiditis (Hashimoto's thyroiditis or Hashimoto's disease) are also conditions that commonly coexist with PBC [2, 14, 16, 22, 24–26, 37, 46, 57, 61].

Floreani et al. [61] reported that, among 361 patients with PBC, 221 (61.2%) had at least one extrahepatic autoimmune disease (follow-up of 8 ± 6.9 years). The authors found a significantly positive association between the female gender and complications by extrahepatic autoimmune conditions in patients with PBC, whereas there were no significant correlations between positive AMA, histological stage, and mean age at diagnosis and PBC with and without extrahepatic autoimmune conditions [61]. Furthermore, they reported that extrahepatic complications of autoimmune diseases did not reduce patient survival, and there were no significant differences between HCC occurrence or extrahepatic malignancy and PBC in patients with and without concomitant extrahepatic autoimmune diseases [61]. Moreover, it has been reported that PBC patients with concomitant SS have a slower disease progression than matched patients with PBC alone [66]. These findings may be useful for the clinical management of SLE patients with concomitant PBC.

### 2.3. Concomitant Occurrence of PBC and SLE

#### 2.3.1. Concomitant Occurrence of PBC in SLE Patients

Several reports indicate that the incidence of coexisting PBC in patients with SLE is ≤2%, with results ranging from 0% to 2.7% [2, 25, 37, 41, 42, 44, 45, 47, 57], although there were differences in the duration of follow-up time in these reports. Moreover, the frequency of PBC in patients with concomitant SLE who have abnormal liver enzyme values or liver dysfunction is reportedly 0%–7.5% [37, 42, 44, 45, 47]. Additionally, no obvious correlations between SLE activity and the incidence of PBC have been reported in patients with SLE [23, 25, 26]. In SLE patients with concomitant PBC, SLE flare-ups are unusual [25, 26].

#### 2.3.2. Concomitant Occurrence of SLE in PBC Patients

Several reports indicated that the incidence of SLE during the follow-up of PBC patients is ≤2%, with the incidence ranging from 0% to 3.7% [2, 16, 17, 20, 25, 61, 67, 68], although there were differences in the duration of follow-up time in these reports. It is unclear whether the incidence of SLE during the follow-up of PBC is significantly higher than that in the general population without autoimmune diseases. However, a large-scale study by Gershwin et al. [14] reported that among 1,032 patients with PBC, 27 (2.61%) also had SLE, and the incidence of SLE in patients with PBC (27/1032, 2.61%) was significantly higher than that in the controls (5/1,041, 0.48%).

#### 2.3.3. Autoantibodies in PBC and SLE Patients

AMAs, particularly M2 antibodies, are useful for serological diagnosis of PBC. Although the percentage of AMA-positive cases in collagen diseases (other than PBC) is low [23, 25, 69], 90%–95% of patients with PBC are AMA-positive [25, 57]. However, patients with AMA-negative PBC exhibit a clinical course similar to their seropositive PBC counterparts [15]. Serological studies of large, presumably healthy cohorts indicated that the prevalence of AMA can be as high as 0.5% [13, 15]. Picceli et al. [70] reported that there was no significant difference in the frequency of AMA positivity between patients with SLE and healthy controls.

Despite this, AMA antibody titers reportedly decrease and undergo negative conversion over time in approximately 1/3 of the SLE patients with concomitant AMA-positive PBC [22, 23, 57, 62]. Matsumoto et al. [57] reported that 2/73 (2.7%) patients with SLE had concomitant PBC; however, both cases were AMA-negative. It may therefore be important to consider AMA-negative PBC in patients with SLE and liver dysfunction. Moreover, anti-double stranded DNA and anti-ribosomal-P antibodies, two serological markers of SLE, were detected in 22% and 5%, respectively, of patients with PBC without other autoimmune diseases [49].

#### 2.3.4. Suspected Common Genetic Susceptibility in Concomitant Cases of SLE and PBC

Recent genome-wide studies have provided an insight into the genetic background of the pathogenesis of autoimmune diseases, including PBC and SLE, and have identified risk loci, such as IRF5-TNPO3, which may be associated with the genetic susceptibility to both SLE and PBC [71–73].

Moreover, osteopontin (OPN), a pleiotropic protein, is important in the immune system signaling, and OPN expression is influenced by the genetic polymorphisms of its promoter, hormones, and cytokines [74]. OPN was reported to be highly expressed in MRL/lpr mice, recognized as one of the spontaneous autoimmune models of SLE [26, 74]. A large number of publications suggested that OPN participates in the pathogenesis of several autoimmune diseases [74], including SLE, and a number of studies demonstrated that an increased plasma concentration, as a result of OPN gene polymorphism and increased protein expression, is associated with SLE susceptibility and/or clinical manifestations of the disease in humans [74, 75]. OPN may also be involved in the susceptibility to PBC [25, 26]. It has been reported that OPN is involved as a chemoattractant cytokine in the recruitment of macrophages and T lymphocytes in liver granulomas, also playing an important role in the production of autoantibodies in PBC [76]. However, although a single-nucleotide polymorphism (SNP) at nt position 9,250 (C–T) in exon 7 in OPN was highly associated with SLE, Kikuchi et al. [77] reported that symptoms and pathologic stages of PBC did not correlate with the variation of this SNP, suggesting no associations between this polymorphism and susceptibility to PBC in Japan.

To our knowledge, these genetic factors were not investigated or not discussed in previously reported cases of concomitant SLE and PBC.

Therefore, it is uncertain whether some patients with concomitant SLE and PBC may have a common genetic susceptibility and/or immunological background favoring the development of these diseases [33].

### 2.4. Cases of Concomitant SLE and PBC

#### 2.4.1. Reported Cases of Concomitant SLE and PBC

Cases of concomitant SLE and PBC retrieved from the English and Japanese scientific literature and Japanese proceedings amount to a total of 34, which are summarized in Table 1 (20 citations in English [17–26] and Japanese [9, 27–32]) and Table 2 (14 Japanese proceedings).

#### 2.4.2. Characteristics of Cases of Concomitant SLE and PBC

Thirty-three of 34 concomitant cases were seen in females. This proportion (97.1%, 33/34) appears high, although PBC and SLE are more common in females. These findings may be in accordance with those reported by Floreani et al. [61], who indicated that there was a significantly positive association between the female gender and the presence of extrahepatic autoimmune conditions in patients with PBC.

Because PBC is more common in middle-aged women and rare in teenagers and because SLE usually affects women of the childbearing age [2, 26], it is assumed that SLE is more likely to be first diagnosed in younger PBC patients with concomitant SLE. However, in the 34 patients with concomitant SLE and PBC, PBC was first diagnosed in 58.8% (20/34) and SLE in 26.5% (9/34); 14.7% (5/34) of the cases were simultaneously diagnosed with SLE and PBC. In the 20 patients in whom PBC first occurred, the interval from the diagnosis of PBC to that of SLE ranged from seven months to 10 years [9, 17, 18, 20–22, 24, 27, 29, 30, 32]. Meanwhile, in nine patients with preceding SLE, PBC was diagnosed 1–19 years after the diagnosis of SLE [19, 23, 25, 26, 31, 32].

Other disease complications in SLE patients with concomitant PBC are listed in Tables 1 and 2. The most common disease presenting concomitantly with SLE and PBC is SjS (23.5%, 8/34), a common complication of both SLE and PBC. Immune thrombocytopenia was diagnosed in three patients (8.8%), and RA, Hashimoto's thyroiditis, and pulmonary hypertension were diagnosed in two patients (5.9%). One patient represented a case of familial PBC [31]. Sato et al. [31] reported a case of a Japanese female who developed asymptomatic PBC at the age of 44, after presenting with SLE. In addition, her father was diagnosed with PBC, indicating a case of familial PBC.

As mentioned above, LC is uncommon in SLE patients with concomitant liver dysfunction. In fact, only one (5%) of 20 SLE patients with concomitant PBC undergoing liver biopsy at the time of PBC diagnosis was found to present with stage IV according to the Scheuer classification, indicating cirrhosis. Similarly, few PBC patients with concomitant SLE develop LC at the time of PBC diagnosis. PBC patients with concomitant SLE who clinically presented with LC (PBC occurred first and SLE subsequently occurred) have also been reported [21].

Ishiguro et al. [9] reported a case of an 81-year-old Japanese female who developed SLE and HCC approximately one year after the diagnosis of PBC. HCC is relatively rare in patients with PBC [9, 59, 60] and, to the best of our knowledge, this is the only case report of PBC with concomitant SLE that concurrently occurs with the development of HCC.

Among the five deaths, two patients presented liver failure secondary to PBC (both were elderly females) [20, 23]. One of these patients died of liver failure two years later, although a liver biopsy at the time of PBC diagnosis indicated that the patient had stage I disease [23]. Liver biopsy of the other patient showed no obvious abnormalities at the time of diagnosis; however, the patient died of liver failure 15 years later [20]. One patient experienced sudden death (unknown etiology) [18]. The other two deaths were attributed to severe conditions: (1) complicated pneumonia and disseminated intravascular coagulation and (2) complicated pneumonia and hemolytic uremic syndrome/thrombotic thrombocytopenic purpura. One of these fatalities was presumably caused by the immunosuppressive condition of the host associated with immunosuppressive therapies.

### 2.5. Cases of Concomitant SLE and Other Autoimmune Liver Diseases

#### 2.5.1. Concomitant of SLE and AIH

The diagnosis of AIH is based on elevated liver enzymes, hypergammaglobulinemia, presence of autoantibodies, and characteristic histological changes [78]. AIH can be divided into two subtypes (type 1 and type 2); type 1 AIH is characterized by the presence of anti-nuclear antibodies (ANA) and/or anti-smooth muscle antibodies, whereas type 2 AIH is characterized by anti-liver/kidney microsome type 1 antibody and/or anti-liver cytosol type 1 antibodies [78]. Coexisting SLE and AIH are rare; the frequency of AIH during the course of SLE appears to range from 2.1% to 3.7% [37, 38, 41, 44, 45, 47, 57]. Teufel et al. [79] reported that the prevalence of additional autoimmune disease in AIH patients was 39.9% (111/278) and the prevalence of SLE was 0.7% (2/278). Patients with AIH sometimes progress to fulminant hepatic failure and advanced LC; hence, differentiating between AIH and lupus hepatitis is critical [47]. However, it is sometimes difficult to discriminate between AIH and lupus hepatitis because immunosuppressive therapy hampers the differential diagnostic considerations, and anti-ribosomal P antibody is not a marker of lupus hepatitis only [38]. Patients with AIH/SLE overlapping or AIH alone also test positive [47]. Moreover, although anti-double stranded DNA antibodies were reported to be specific for SLE, these are also common in patients with ANA-positive type 1 AIH [80]. Further, the diagnostic criteria of SLE do not appear useful for discriminating AIH from lupus hepatitis [81]. Therefore, histological examination of the liver is instrumental in establishing the differential diagnosis between lupus hepatitis and AIH [51, 81, 82]. Periportal piecemeal necrosis associated with lobular activity, rosetting of liver cells, or dense lymphoid infiltrates are prominent in AIH, whereas in SLE inflammation is usually lobular and occasionally periportal with a paucity of lymphoid infiltrates [82].

It should be noted that a higher incidence of AIH is seen in patients affected by juvenile-onset SLE [41, 45, 48, 78].

#### 2.5.2. Concomitance of SLE and PSC

PSC is also a cholestatic liver disease associated with autoimmune processes [58]. Although PBC and PSC are associated with chronic cholestatic liver disease, there are many clinical and epidemiological differences [58]. PSC is best known for its hepatobiliary manifestations accompanied by ulcerative colitis. Additionally, the prevalence of SLE patients with concomitant PSC appears to be extremely rare. To the best of our knowledge, only four reports are available on SLE patients with concomitant PSC to date [3, 83–85]. One of the reasons for the scarcity of reported cases is the low prevalence of PSC itself, which ranges from 4 to 16 per million [86]. Conversely, the reported prevalence of PBC is 19–402 per million [13, 15, 34]. Whether SLE with concomitant PSC occurs by chance or whether these entities have a common immunological basis remains unclear.

## 3. Conclusions

In this paper, 34 cases of SLE with concomitant PBC published in English and Japanese, including Japanese proceedings, were reviewed and summarized. Of the concomitant cases, 97.1% (33/34) were represented by females, and PBC was diagnosed first in 69.0% (20/29), except for five patients in whom both SLE and PBC were almost simultaneously diagnosed. The most common autoimmune disease present in SLE patients with concomitant PBC was SjS (23.5%, 8/34). Moreover, in SLE patients with concomitant PBC, no death cases resulting from the aggravation of the initially diagnosed disease that occurred soon after the onset of the subsequent disease have been reported.

However, it remains uncertain whether concomitant cases occur by chance or share a common immunological or genetic basis. Further studies are warranted to better understand these concomitant autoimmune diseases.

## Figures and Tables

**Table 1:** Characteristics of 20 systemic lupus erythematosus patients with concomitant primary biliary cirrhosis derived from the literature in English and Japanese.

Case	Sex	Age at diagnosis of SLE (years)	Age at diagnosis of PBC (years)	PBC prior to SLE	Remarks	References
1	F	39	33	+		[17]
2	F	58	53	+		[18]
3	M	53	50	+		[18]
4	F	39?	35	+	Sudden death (etiology?)	[18]
5	F	25	29	−	Lupus nephritis (renal failure)	[19]
6	F	60	53	+	Liver failure	[20]
7	F	65	64?	+		[21]
8	F	41?	37	+		[22]
9	F	54	72	−	Liver failure	[23]
10	F	57	47	+		[24]
11	F	21	29	−		[25]
12	F	69	70	−		[26]
13	F	63 or 64	62	+	RA and Sjögren's syndrome	[27]
14	F	41	41	Sim	Immune thrombocytopenia	[28]
15	F	34 or 35	31	+	Lupus nephritis	[29]
16	F	48	40	+	Sjögren's syndrome	[30]
17	F	27	44	−	Familial PBC case	[31]
18	F	46?	65	−	Sjögren's syndrome	[32]
19	F	55	52?	+	Sjögren's syndrome	[32]
20	F	81	80	+	Hepatocellular carcinoma	[9]

SLE: systemic lupus erythematosus; PBC: primary biliary cirrhosis; F: female; M: male; Sim: simultaneous; RA: rheumatoid arthritis.

**Table 2:** Characteristics of 14 systemic lupus erythematosus patients with concomitant primary biliary cirrhosis derived from Japanese proceedings.

Case (Year)	Sex	Age at diagnosis of SLE (years)	Age at diagnosis of PBC (years)	PBC prior to SLE	Remarks
1 (1984)	F	33?	28	+	Immune thrombocytopenia and pulmonary hypertension
2 (1987)	F	41	41	Sim	
3 (1991)	F	51	50	+	Immune thrombocytopenia
4 (1993)	F	?	46	−	Sjögren's syndrome
5 (1993)	F	51	51	Sim	
6 (1996)	F	39	46	−	Hashimoto's thyroiditis
7 (1999)	F	46	39	+	RA, Sjögren's syndrome, and lupus nephritis (death)
8 (2000)	F	51	48	+	Sjögren's syndrome
9 (2001)	F	45	51	−	Lupus nephritis
10 (2003)	F	48	47	+	HUS/TTP, DIC, and pneumonia (death)
11 (2005)	F	54	54	Sim	
12 (2008)	F	59<	?	+	Sjögren's syndrome and pulmonary hypertension
13 (2013)	F	64	64	Sim	
14 (2013)	F	65	64	+	Hashimoto's thyroiditis and lupus nephritis

SLE: systemic lupus erythematosus; PBC: primary biliary cirrhosis; F: female; M: male; Sim: simultaneous; RA: rheumatoid arthritis; HUS/TTP: hemolytic uremic syndrome/thrombotic thrombocytopenic purpura; DIC: disseminated intravascular coagulation.
